# Targeting Matrix Stiffness and Mechanotransduction in Breast Cancer: Implications for Emerging Therapies

**DOI:** 10.3390/ijms27031510

**Published:** 2026-02-03

**Authors:** Michael Hall, Ozichi Amobi, John Khalaf, Afees John Olanrewaju, Eileen Brantley

**Affiliations:** 1 Department of Basic Sciences, Loma Linda University Health School of Medicine, Loma Linda, CA 92354, USA; mhhall@students.llu.edu (M.H.); oamobi001@ucr.edu (O.A.); jpkhalaf1360@gmail.com (J.K.); 2 Eureka Research Laboratory, Department of Anatomy, Babcock University, Ilishan-Remo 121103, Ogun State, Nigeria; olanrewajuj@babcock.edu.ng; 3 Department of Pharmaceutical Sciences, Loma Linda University Health School of Pharmacy, Loma Linda, CA 92354, USA; 4 Center for Health Disparities and Molecular Medicine, Loma Linda University Health School of Medicine, Loma Linda, CA 92354, USA

**Keywords:** extracellular matrix stiffness, mechanotransduction, breast cancer, epithelial-mesenchymal transition, tumor invasion, metastatic cascade, disparities

## Abstract

Breast cancer remains a leading cause of mortality among women worldwide. The inherent heterogeneity in tumors among patients with breast cancer poses a challenge to effective therapeutic management. The extracellular matrix (ECM) is an important structural component of the tumor microenvironment (TME) that regulates cellular behavior. When the ECM adopts a stiff configuration, this coincides with biochemical remodeling in response to biomechanical cues that drive tumor cell invasion, immune evasion, and metastatic spread in breast cancer. Emerging studies suggest that patient ancestry significantly impacts ECM stiffness to contribute to disparities in breast cancer survival. In this review, we discuss recent advances in our understanding of how the tumor ECM orchestrates breast cancer invasion and metastasis through mechanotransduction signaling to promote breast cancer progression. We also discuss ancestry-associated differences in breast ECM architecture and agents targeting mechanotransduction signaling pathways with potential to treat breast cancer and improve patient outcomes. Collectively, this review will highlight the significance of tumor mechanobiology and present emerging therapies that target stiffness-sensitive mechanotransduction pathways. By integrating mechanistic insights with therapeutic innovation, we aim to support the development of ECM-targeted therapies to enable more efficacious treatment of aggressive breast cancer subtypes.

## 1. Introduction

Cancer remains the leading cause of morbidity and mortality worldwide. Globally, more than 600,000 women lose their lives to breast cancer each year. Metastasis can be attributed to most cancer-related deaths. Invasion and metastasis, hallmarks of cancer, represent complex processes that allow cancer cells to escape their primary site, survive in circulation, and colonize other organs [1]. During local invasion, tumor cells commonly lose their epithelial traits through downregulation of E-cadherin and upregulation of vimentin or N-cadherin characteristic of epithelial-to-mesenchymal transition (EMT) [2,3,4]. This shift enhances cell motility, enabling tumor cells to detach from the primary mass. These processes are not driven solely by cell-autonomous mechanisms but also by the tumor microenvironment (TME). The extracellular matrix (ECM) is a network of proteins and polysaccharides that provides structural and biochemical support to tissues within the TME along with an array of diverse cell types. The ECM actively participates in tumor progression, invasion, and metastasis and is now recognized as a highly bioactive structure that promotes mechanotransduction signaling [5]. In breast tumors, the ECM composition and mechanical properties are altered by both physical and biochemical cues, which modulate tumor cell plasticity, immune evasion, and the formation of a metastatic microenvironment. In this review, we will discuss how relative ECM stiffness activates mechanotransduction signaling to promote breast cancer progression, ancestry-associated differences in breast ECM architecture, and agents that target mechanotransduction signaling with the potential to treat breast cancer and improve patient outcomes.

## 2. The Dynamic Tumor ECM

### 2.1. Normal Versus Cancer Breast ECM Organization

**Figure 1:**
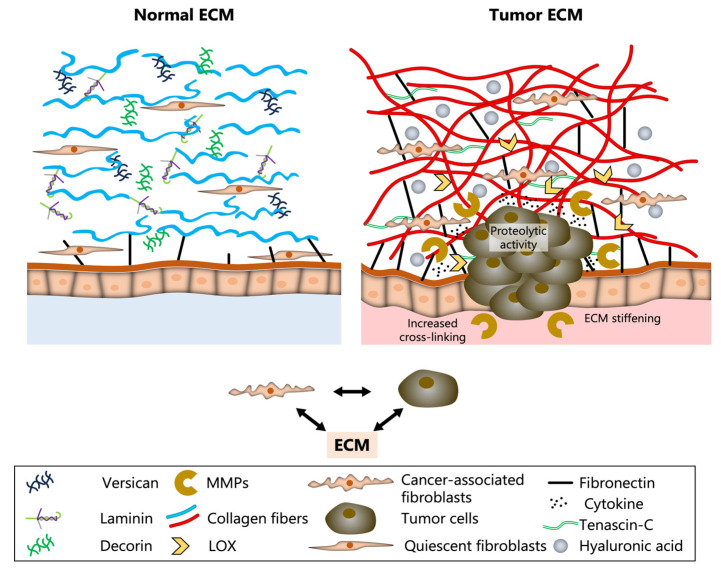
Normal versus tumor structural and biochemical composition of the ECM. The normal ECM exhibits organized collagen fibers, distributed glycoproteins (e.g., laminin, versican), and quiescent fibroblasts, maintaining tissue homeostasis. In contrast, the tumor ECM is characterized by dense, disorganized collagen, enhanced crosslinking by LOX, increased matrix metalloproteinase (MMP) activity, and infiltration of cancer-associated fibroblasts (CAFs). These changes contribute to ECM stiffening, altered cytokine signaling, and proteolytic remodeling, which facilitate tumor invasion, migration, and progression. Key matrix components and cell types are depicted in the legend above.

The ECM is a complex network of proteins and polysaccharides. The most notable of these proteins is collagen, which makes up approximately 30% of the body’s proteins and is a key component of the ECM [6]. It is the most abundant protein in the human body, providing tensile strength and structural integrity to tissues and forming a critical backbone of the ECM. In healthy breast tissues, the ECM maintains homeostasis by supporting proper tissue function and intercellular communication. The architecture of normal breast ECM consists of collagen fibers, laminin, fibronectin, and proteoglycans such as decorin and versican, arranged in a supportive, balanced network (Figure 1) [6]. Quiescent fibroblasts are dispersed throughout the matrix, maintaining its homeostasis and ensuring the proper tissue function within the environment [7]. The basement membrane is an organized layer that further reinforces epithelial integrity and polarity. A soft or compliant ECM is characterized as disorganized and more amenable to adjustments.

In breast cancer, the ECM undergoes significant remodeling to alter its composition, structure, and mechanics, leading to malignant progression and metastases. Under these circumstances, the ECM becomes stiffer as tumor-promoting proteins increase. These changes can be attributed in part to cancer-associated fibroblasts (CAFs), tumor cells, and matrix-modifying enzymes, which together reshape and reorganize the TME [7]. By activating mechanotransduction pathways, promoting matrix degradation, and releasing bioactive fragments, the tumor ECM promotes an invasive phenotype with immune infiltration [4]. The ECM regulates invasion and metastasis, two processes that are among the most lethal aspects of cancer progression. The ECM plays an evolving role in cancer biology. Specifically, ECM-mediated dysregulation often activates mechanotransduction pathways promoting tumor invasion and metastasis (Figure 1). During remodeling, the ECM becomes densely packed with the increased deposition and cross-linking of collagen fibers, accumulation of fibronectin and Tenascin-C, and elevated levels of hyaluronic acid [6]. Cancer-associated fibroblasts (CAFs) and tumor cells actively contribute to this altered matrix by secreting matrix metalloproteinases (MMPs) and lysyl oxidase (LOX), enzymes that degrade and stiffen the ECM [7]. This causes a cascade of changes within the TME that not only supports breast cancer cell proliferation and invasion but also impedes immune cell infiltration and therapeutic delivery. Dynamic, reciprocal interactions occur among stomal cells, tumor cells, and the ECM. This remodeling process fundamentally alters the tissue’s biochemical and biophysical properties, setting the stage for enhanced tumor progression, invasion, and metastasis.

### 2.2. Tumor Associated ECM Remodeling

The ECM in breast tumor tissue undergoes substantial structural and compositional changes compared to normal breast ECM (Figure 1). Tumor-associated ECM is characterized by increased density, organization, and biochemical complexity, all of which contribute to a microenvironment that actively promotes breast cancer progression. One of the most notable features of the breast tumor ECM is the deposition and cross-linking of collagen fibers, illustrated by a thick, red, tangled network (Figure 1) [6]. This accumulation results in increased tissue stiffness, which not only provides a place for tumor cell invasion but also activates the mechanotransduction pathways that further promote malignant behavior. Alongside collagen, there is notable upregulation of glycoproteins such as fibronectin and tenascin-c, as well as elevated levels of hyaluronic acid depicted as gray spheres [6]. These changes disrupt the normal ECM architecture creating a more permissive environment for the tumor’s growth and dissemination.

The cellular composition of the ECM is impacted and altered within breast tumors. Quiescent fibroblasts, which help to maintain ECM homeostasis in normal tissue, are replaced or supplemented by cancer-associated fibroblasts (CAFs) [7]. These fibroblasts, shown as elongated cells with distinct morphology, are major producers of ECM components and remodeling enzymes, along with tumor cells, which help secrete high levels of matrix metalloproteinases (MMPs) and Lysyl oxidase (LOX), represented by the yellow and gold icons (Figure 1) [7]. MMPs degrade ECM barriers to facilitate tumor cell migration, while LOX enzymes catalyze collagen cross-linking, thereby increasing matrix stiffness [6,8,9]. Remodeling of the ECM leads directly to the growth of the tumor and becomes a driver of malignancy. The altered ECM composition and structure provide signals, both mechanical and biochemical, that promote cancer cell invasion, immune evasion, and metastatic potential. The communication between CAFs, tumor cells, and the ECM, as depicted in Figure 1, conveys the complexity of the TME and its central role in cancer invasion and metastasis [7].

### 2.3. Dynamic Remodeling of the ECM in Breast Cancer Progression

The ECM in breast cancer is subject to dynamic change and remodeling that alters the TME and drives malignant progression. Quantitative and qualitative changes in ECM composition, structure, and function characterize this process (Figure 1). In breast tumors, ECM components such as collagen, fibronectin, and tenascin-C are produced in excess by CAFs and, to a lesser extent, by the tumor cells themselves [7]. The tumor ECM promotes the overproduction of a dense, tangled network of collagen fibers (red) and increased fibronectin (black lines) [6]. Furthermore, the ECM is actively degraded by MMPs (gold “C” shapes). CAFs and tumor cells secrete MMPs [7]. MMPs break down structural barriers, releasing bioactive fragments and growth factors that further stimulate tumor cell migration and invasion [8,9].

A hallmark of tumor ECM remodeling is the increased cross-linking of collagen fibers, mediated by lysyl oxidase (LOX, yellow arrows) [6]. This enzymatic activity results in a stiffer matrix, as shown by the thickened, aligned collagen bundles in the figure [6]. ECM stiffening is not merely a structural change; it also directly influences behavior by activating mechanotransduction pathways that promote proliferation, survival, and motility of cancer cells [4,10,11]. Altered cellular interactions promote dynamic remodeling of the ECM, reshaping the interactions among various cell types within the TME. The arrows in Figure 1 indicate the reciprocal communication between CAFs, tumor cells, and the ECM [7]. CAFs respond to signals from the tumor cells by producing more ECM proteins and remodeling enzymes, while the altered ECM, in turn, provides signals that reinforce the activated, pro-tumorigenic state of both CAFs and cancer cells [7].

Finally, these remodeling processes create an ECM that is dense, heterogeneous, and highly permissive to cancer cell invasion. The remodeled ECM not only facilitates local tissue invasion and migration but also supports the escape of tumor cells into the circulation and their eventual colonization of distant organs. Additionally, the altered ECM can act as a barrier to immune cell infiltration and to the entry of therapeutic agents, contributing to immune evasion and treatment resistance. In summary, dynamic ECM remodeling is a central driver of cancer progression, transforming the TME into a landscape that actively supports invasion, metastasis, and therapy resistance [7].

## 3. Ancestry-Associated ECM Differences

While breast cancer survival has steadily improved in the US, women of African ancestry are less likely to survive breast cancer as compared to women of other ancestries [12]. Disparities in breast cancer survival persist even after accounting for differences in access to care and socioeconomic conditions in part due to a higher incidence of more aggressive subtypes, particularly among those under the age of 50 [13]. It is now appreciated that differences in tumor biology based on genetic ancestry contribute to breast cancer disparities [14,15].

Ancestry-associated differences in the TME often lead to differential response to therapy and disparities in clinical outcomes [16,17,18]. Emerging data suggest that differences exist in the stroma and ECM, including collagen organization within tumors, which lead to disparate outcomes when comparing women of different ancestries [19,20,21]. These recent studies have helped to increase our understanding of how ancestry-linked variations in ECM structure impact tumor progression and therapeutic response in breast cancer.

### 3.1. ECM Composition and Stromal Programs

The ECM composition can become altered and collaborate with the stroma within the TME to influence breast cancer growth in an ancestry-associated manner. Recent studies have identified a stromal cell population expressing PROCR, ZEB1, and PDGRFRα (PZP cells), which are abundant in normal breast tissue from women of African ancestry (AA) but predominantly located in tumor-adjacent stroma in women of European ancestry (EA) [19]. Functional studies indicated that PZP cells facilitate leader–follower epithelial invasion by promoting fibronectin deposition and activating AKT signaling in epithelial cells. Pharmacological inhibition of AKT diminished this invasive phenotype, underscoring ancestry-associated predispositions in stromal behavior [19]. Ancestry-specific stromal programming variations, including fibronectin-enriched stroma, may predispose women of African ancestry to more aggressive tumor biology.

### 3.2. Collagen Density and Organization 

Emerging data that suggest ancestry-associated variations in the collagen structure and organization may impact clinical outcomes in breast cancer. A pilot study of breast tumor samples from African American and Caucasian patients with triple negative breast cancer (TNBC), which lacks hormone and human epidermal growth factor 2 receptors, using machine learning, revealed differential EMT-associated gene expression based on ancestry that was related to the extracellular matrix [22]. They show that a stiffer surrounding matrix can alter the tumor structure and transform the cancer into a more aggressive form. More recently, second-harmonic generation (SHG) microscopy identified collagen fiber architecture consistent with a stiff ECM phenotype in TNBC tissue samples from patients of African ancestry to a greater extent than those of European ancestry [21]. Investigators in this study found that closely curved fibers tend to be located at the tumor periphery, promoting cancer cell movement and dispersion.

### 3.3. Arrangement of Collagen Fibers and Mammogram Density

Emerging studies suggest that the arrangement and density of ECM fibers may differ by ancestry, particularly among patients with TNBC. Tumors from patients of African ancestry exhibited markedly dense and intricate collagen fiber networks, characterized by increased fiber length, branching points, and fractal dimension, along with reduced lacunarity compared to tumors from patients of European ancestry [21]. These macro-architectural features revealed collagen characteristics associated with an aggressive TNBC phenotype and correlated strongly with invasive potential and a poorer prognosis [6].

Mammography density, frequently used as a proxy for ECM abundance and collagen content, has been reported among patients of diverse ancestries, though the findings are not always consistent. Women with very dense breasts (>75% dense area in the breast) have a four to six-fold increased risk of breast cancer as compared to women with low-density (<5–10% dense area) breasts [23,24,25]. High-density breasts contain a higher proportion of epithelial and fibrous tissues with significantly higher tissue rigidity [26] due to ECM deposition and remodeling by fibrous tissues. High mammography density is associated with increased breast cancer risk among women of African Ancestry [27,28,29,30], likely due to genetic variations [31]. Whether high mammography density among women of African Ancestry contributes to poor TNBC outcomes is yet to be fully elucidated.

### 3.4. Enzymatic Crosslinking and Remodeling Activity 

Researchers investigating the mechanism of action of the surrounding tissue (ECM) have discovered that additional enzymes, such as lysyl oxidase (LOX) and matrix metalloproteinases (MMPs), have various effects. Differences in breast tumor morphological and breast tumor architecture between patients of European and African ancestry were captured using artificial intelligence and machine learning approaches to suggest that ancestry-associated differences in the ECM contribute to breast cancer disparities [20]. This study discovered that tumors from patients of African ancestry were more likely to exhibit elevations in LOX. One study found that patients with breast cancer of African ancestry were also more likely to have tumors with high MMP activity, believed to contribute to a more aggressive phenotype compared to those of European ancestry [32]. On the other hand, a more recent multiethnic study did not discover significant increases in breast cancer risk based on ancestry due to differences in MMP plasma levels [33]. This suggests that elevations in MMP levels may occur following the onset of aggressive breast cancer, which is seen more often among women of African ancestry. LOX aids in binding collagen fibers, making the ECM more difficult to degrade. MMPs degrade ECM components to facilitate cell movement. Thus, elevations in LOX and MMP promote a stiff ECM, which in turn enables breast cancer cell invasion and proliferation. 

### 3.5. Immune ECM Interactions 

The ECM influences immune cell function within the TME. Emerging evidence suggests that the tumor immune microenvironment plays a role in breast cancer disparities. For instance, patients of African ancestry are more likely to possess higher plasma levels of the pro-inflammatory cytokine interleukin-6 and the adipocyte-derived cytokine resistin associated with reduced breast cancer survival [34,35]. The quantity and the nature of the immune cells present in breast cancers have been shown to vary by ancestry [18,36]. Women of African ancestry are more likely to exhibit a stronger immune response, characterized by higher cytokine gene expression and increased infiltration of M1 macrophages [37]. The stroma within basal-like tumor subtypes has previously been shown to exhibit greater aggressiveness and invasion, coinciding with increased infiltrating macrophages [38]. This suggests that breast cancer invasion and aggression are associated with ECM stiffness and immune cell infiltration, and this relationship may explain the increased aggressiveness found among patients of African ancestry diagnosed with TNBC. Immune cells in other tissues face more rigid barriers in the ECM and distorted collagen meshwork, which may hamper their entry into tumor cells and even reduce immune surveillance. These differences in immune TME may explain why patients of diverse ancestries exhibit differential responses to immunotherapies [18].

## 4. ECM-Mediated Mechanisms of Invasion and Metastasis-Biochemical Signaling

**Figure 2:**
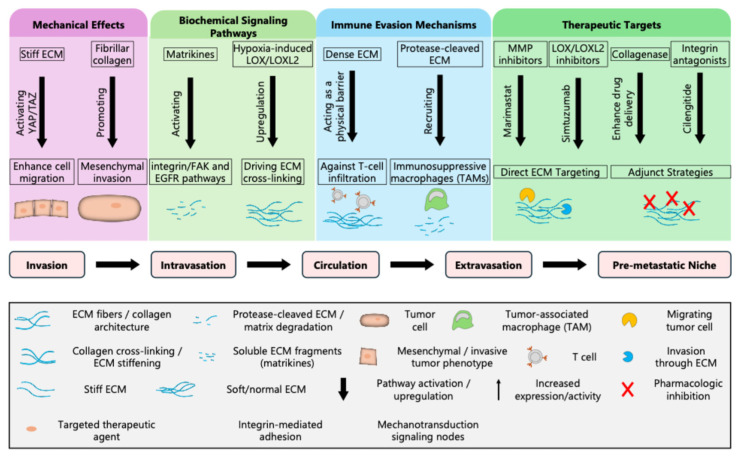
ECM remodeling entails multiple processes amenable to therapeutic targeting. Mechanical effects (purple) from ECM stiffening activate biochemical signaling pathways to enhance cell migration and mesenchymal invasion. These biochemical signaling or mechanotransduction pathways (light green), such as Integrin/FAK and EGFR pathways, respond to matrix cues mediated by lysyl oxidase (LOX) and other matrisome proteins. The immune evasion mechanism (blue) represents an important consequence of ECM remodeling and activation of mechanotransduction signaling. Dense and proteolytically remodeled ECM impairs T cell infiltration and recruits immunosuppressive macrophages. Finally, therapeutic targets (dark green) are highlighted, including MMP inhibitors, LOX/LOX2 inhibitors, and integrin antagonists, offering plausible strategies to disrupt ECM-mediated tumor progression. The ECM contributes to each phase of metastasis from invasion to pre-metastatic niche formation (bottom row).

ECM remodeling in cancer alters the TME at the cellular level enhancing invasion and metastasis. As seen in Figure 2, remodeled ECM contributes to metastasis through mechanical alterations, biochemical signaling, and immune evasion. This remodeling provides specific opportunities for therapeutic intervention. Remodeled ECM components, including matrikines, activate signaling pathways such as integrin/FAK and EGFR pathways, both of which promote tumor cell migration, invasion, and survival [4,39,40,41,42]. Hypoxia within the TME increases the expression of enzymes such as LOX, which promotes mechanotransduction pathways, thereby enhancing breast cancer migration [43]. These coordinated biochemical cues collectively create an environment more permissive for cancer cell movement and dissemination.

### 4.1. Mechanical Effects and Immune Evasion

The tumor ECM is characterized by increased stiffness and highly aligned fibrillar collagen [6]. These mechanical changes are sensed by cancer cells through mechanotransduction pathways [4,10]. For instance, the YAP/TAZ activation pathway enhances cell migration and supports the transition to a more invasive, mesenchymal phenotype [44]. A stiff ECM not only physically supports cell movement but also reinforces invasive signaling networks within tumor cells. This disrupted, and dysfunctional ECM acts as a physical barrier, impeding the infiltration of cytotoxic T cells and other immune effectors [45]. In addition, protease-cleaved ECM fragments help to recruit immunosuppressive macrophages (TAMs), which further dampen anti-tumor immune responses. These immune evasion strategies allow tumor cells to escape immune surveillance and establish metastatic colonies.

### 4.2. The Metastatic Cascade: From Local Invasion to Colonization

**Figure 3:**
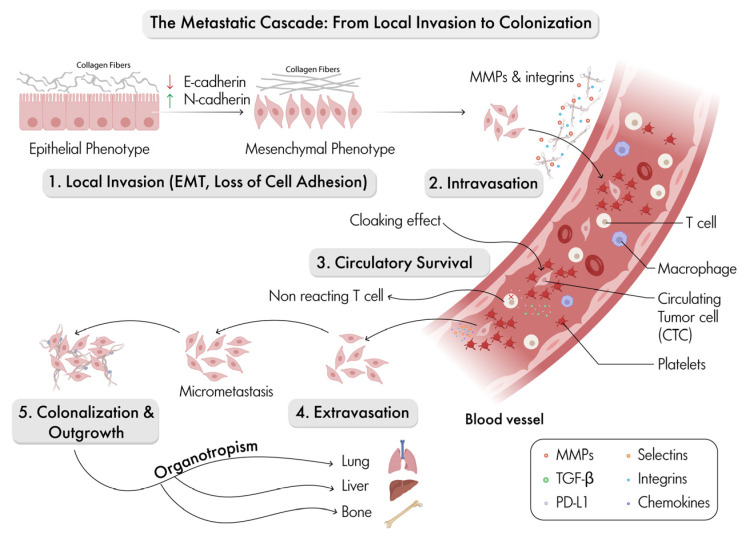
Overview of the sequential steps in the metastatic cascade. Epithelial cells undergo epithelial–mesenchymal transition (EMT) and lose cell-to-cell adhesion to initiate local invasion. ECM-associated molecules, such as matrix metalloproteinases (MMPs) and integrins support intravasation and survival in the circulation, where circulating tumor cells (CTCs) evade immune surveillance. The metastatic process continues with extravasation into secondary sites culminating in organ-specific colonization and outgrowth (organotropism). Key signaling molecules, immune modulators, and ECM components that interact to regulate each phase are annotated, highlighting the interplay between tumor cells and the tumor microenvironment. Symbols defined: ↑ depicts increase; ↓ depicts decrease.

Figure 3 provides a schematic of the steps involved in the metastatic cascade. Specifically, it provides an overview of both the physical transitions and molecular mediators that allow tumor cells to travel from the primary site and establish secondary growths in distant organs. The process starts with local invasion where epithelial tumor cells undergo EMT transition (EMT) [4]. This occurs via downregulation of E-cadherin and upregulation of N-cadherin [3,4]. This shift to a mesenchymal phenotype is driven by transcriptional programs and TME cues, leading to the loss of cell-to-cell adhesion and increased motility [7]. Matrix metalloproteinases (MMPs) and integrins facilitate ECM degradation, allowing cells to breach tissue boundaries [4,8,9].

After intravasation, tumor cells enter the vasculature, where their interactions with endothelial cells and ECM remodeling—mediated notably by integrins and MMPs—are highly critical [4,8,9]. Once in the bloodstream, circulatory survival becomes a major challenge for circulating tumor cells (CTCs), which must evade immune surveillance and withstand shear stress. Platelet cloaking and suppression of T cell responses, as illustrated in Figure 3 [4], support this survival. Furthermore, macrophages and other immune cells in the blood can either promote CTC survival or facilitate their clearance [4].

Extravasation is the process by which CTCs adhere to and travel to the endothelium at distant sites. This step is regulated by selectins, integrins, chemokines and endothelial remodeling, enabling tumor cells to exit the circulation and invade new tissue environments [46,47,48]. Once in the new tissue, tumor cells may remain dormant as micro-metastases or undergo mesenchymal-to-epithelial transition (MET) to form overt secondary tumors [49]. Finally, colonization and outgrowth are influenced by local microenvironmental factors, immune interactions, and the tumor cells’ ability to adapt to the new environment [4,7]. Organotropism is the tendency of certain cancers to metastasize to specific organs such as the lung, liver, and bone, guided by molecular cues and the compatibility of the metastatic environment [6]. Throughout the cascade, molecular mediators such as MMPs, TGF-β, PD-L1, selectins, integrins, and chemokines orchestrate the transitions, supporting tumor cell survival, migration, immune evasion, and colonization [4,8,9].

### 4.3. Therapeutic Targets and Integration Within the Metastatic Cascade

As summarized in the lower panel of Figure 2, ECM-driven mechanisms are interwoven and linked to each stage of the metastatic cascade. Considering the central role of ECM remodeling in metastasis, several therapeutic strategies are arising to help target these processes. Notably, a more direct way to target ECM is to use MMP inhibitors and LOX/LOXL2 inhibitors as they can disrupt ECM degradation and cross-linking. Adjunct strategies, such as collagenase, can enhance drug delivery by breaking down dense ECM, while antagonists can interfere with cell-ECM signaling to inhibit invasion and metastasis [50]. Each step from local invasion and intravasation, circulation and extravasation, to the establishment of pre-metastatic niches in distant organs offers potential sites for therapeutic intervention and improved patient outcomes. Figure 2 provides a comprehensive visual of ECM remodeling with a roadmap of how its remodeling orchestrates the complex, multi-step process of cancer invasion and metastasis. This highlights both the underlying biology and the avenues for clinical translation.

There are several interconnected signaling networks that mediate cellular responses to ECM stiffness, topography, and tensile forces. Some of the established pathways include the Yes-associated protein (YAP) and transcriptional co-activator with PDZ-binding motif (TAZ), the ERK/RSK1/TWIST1 pathway, and other integrin-mediated pathways (e.g., β-1 integrin and β-6 Integrin).

The YAP/TAZ pathway functions downstream to the Hippo signaling pathway [44]. Increased ECM stiffness leads to the inactivation of the core Hippo kinase cascade (MST1/2 and LATS1/2), which turns off the Hippo signaling pathway [51]. In the inactive Hippo signaling pathway, the YAP/TAZ complex is dephosphorylated, allowing it to translocate to the nucleus. In the nucleus, the unphosphorylated YAP/TAZ complex binds to a transcriptional enhanced-associate domain (TEAD) [52]. TEAD then switches from a repressor state to an activator state, driving the expression of genes involved in EMT, cancer cell formation, proliferation, and invasion.

The Rho-associated coiled-coil-containing protein kinase (ROCK)-myosin IIA-filamentous actin (F-actin) pathway contributes to immunosuppression in the TME [53]. ECM stiffening activates the Rho-ROCK-myosin-IIA-F-actin pathway. This signaling cascade promotes the organization of NMHC-IIA-F-actin stress fibers, which then interact with tripartite motif-containing protein 14 (TRIM14), preventing it from binding to cGAS (cyclic GMP-AMP synthase) [53]. Without TRIM14 binding, cGAS is targeted for autophagic degradation, ultimately resulting in reduced tumor immunogenicity and a suppression of the anti-tumor immune response [53].

The ERK/RSK1/TWIST1 signaling cascade plays a critical role in regulating EMT and metastasis in cancer cells [54]. Increased ECM stiffness triggers the phosphorylation of the Ephrin type-A receptor 2 (EPHA2) on the surface of cancer cells, which then binds to and activates LYN kinase [40]. LYN kinase triggers the MAPK/ERK signaling cascade, which leads to the phosphorylation and activation of ribosomal S6 kinase 1 (RSK1) [55]. RSK1 phosphorylates sites on the transcription factor TWIST1 to facilitate its nuclear localization. In the nucleus, TWIST1 promotes the transcription of genes that drive EMT and cancer cell metastasis.

Integrins are cell adhesion proteins and transmembrane heterodimers consisting of α and β subunits. All except α6β4-integrin mediate interactions between the cytoskeleton and the ECM. Integrins cooperate with other signaling pathways to promote aberrant growth of breast cancer cells, often once ECM proteins, including collagen, fibronectin, and laminin, bind to their integrin receptors [56]. There are multiple α/β heterodimers that help mediate integrin signaling. For instance, β1-integrin heterodimerizes with α6-integrin, and this complex plays a prominent role in breast cancer progression and ECM-mediated mechanotransduction. Integrin-β6 is often coupled with α5-integrin, and this heterodimer cooperates with growth factors to promote cancer cell growth while α5-β6-integrin expression is lower in healthy tissue than in tumor tissue [57]. It is primarily expressed in breast cancer in late stages, characterized by a more invasive phenotype [58,59]. While EMT transition has been evident with high β6-integrin levels for colon cancer, this has not been clearly shown for breast cancer [60].

### 4.4. Mechanobiology- Driven Strategies to Address ECM Stiffness-Mediated Abnormalities

Though precision oncology historically implements strategies tailored to target mutations that drive breast cancer progression, mechanobiology-driven precision therapeutic approaches have the potential to benefit a subset of patients who currently lack efficacious treatment options. The ECM provides structural support for the cell, regulates cell growth, and promotes intracellular communication. Increases in ECM stiffness drive breast cancer progression [38,40,61]. In particular, high ECM stiffness promotes ligand-independent phosphorylation of EPHA2, enabling the recruitment and activation of LYN kinase, which in turn phosphorylates the EMT transcription factor TWIST [40]. TWIST partners with G3BP2 to translocate from the cytosol to the nucleus to promote EMT, invasion, and metastases [40]. In general, ECM stiffness is higher in breast cancer tissue than in normal tissue and acts as a barrier to drug delivery [62]. So, targeting ECM stiffness directly or via mechanotransduction signaling pathways activated by enhanced ECM stiffness is expected to help thwart breast tumorigenesis and increase patient survival.

The propensity for a stiff ECM to drive cancer progression via mechanotransduction activation has opened the door to many new avenues for therapeutic intervention. Targeting ECM remodeling is a new, promising strategy to disrupt the metastatic cascade, enhance the efficacy of existing treatments, and, theoretically, improve patient outcomes. Several therapeutic approaches are being investigated to interfere with biochemical and mechanical pathways by which the remodeled ECM facilitates invasion, immune evasion, and metastatic niche formation (Figure 2) [62,63]. These approaches often affect cancer-associated fibroblasts (CAFs) or modulate ECM component production within the TME [7].

### 4.5. Novel Agents That Target Mechanotransduction Pathways: Preclinical Studies

Mechanotransduction signaling involves a process by which cells sense and respond to mechanical cues from the ECM. Mechanotransduction signaling is an important regulator of tumor progression and therapeutic response in breast cancer. Agents that target mechanotransduction signaling pathways offer a promising strategy to inhibit tumor growth, reverse drug resistance, and enhance anti-tumor immunity. They can modulate biochemical signals arising from biomechanical cues emanating from the ECM of breast cancer cells and ultimately help improve clinical outcomes.

#### 4.5.1. Integrin Inhibitors

Preclinical studies have sought to evaluate integrin antagonists as potential therapies for breast cancer, most often in combination with established anticancer agents. While β1 integrin has been implicated in ECM-mediated breast progression, a study found that targeting this integrin can markedly promote lung metastases in TNBC models, limiting its clinical application in this particular breast cancer subtype [64]. In another study, α5β3-integrin, in combination with docetaxel, demonstrated proof of concept as a strategy to enhance docetaxel efficacy using nanoparticle-based delivery to counteract breast cancer metastases [65]. These investigators also found that a α5β3-integrin nanoparticle system in conjunction with a c-myc inhibitor effectively reduced protumor macrophages and enhanced antitumor macrophage activity in preclinical models of breast cancer [66].

Cilengitide, an α5β3-integrin and α5β5-integrin antagonist helps to disrupt the interactions between tumor cells and the ECM in cancer [67]. Cilengitide inhibits cell adhesion, migration, and survival signals that are essential for metastasis. Although cilengitide went into clinical trials as an agent to treat aggressive brain tumors such as glioblastoma, it failed to meet endpoints and thus remains an investigational agent. Cilengitide has demonstrated the capacity to block metastatic breast cancer progression [68,69,70], radiosensitize breast cancer cells [71], and enhance radioimmunotherapy in breast cancer xenografts [72]. Cilengitide can restore sensitivity to paclitaxel following the onset of EMT transition and paclitaxel resistance in breast cancer cells [73]. Importantly, β3-integrin confers resistance to trastuzumab, and thus cilengitide can also resensitize trastuzumab-resistant HER2-positive breast cancer cells [74]. More recently, cilengitide has been shown to block pericardial calcification and blunt breast cancer progression when used in combination with an insulin-like growth factor receptor 1 antagonist [75].

Integrin pathways help to activate TGFβ during ECM remodeling [76]. A recent preclinical study demonstrated greater efficacy with a bifunctional fusion protein targeting both the TGFβ pathway and programmed death-ligand 1 (PD-L1) [77]. EMT, ECM deposition, and fibrosis were all reduced.

#### 4.5.2. Matrix Metalloproteinase Inhibitors

A key area of focus is the inhibition of ECM-degrading enzymes, particularly matrix metalloproteinases (MMPs) [8,9]. MMPs play a critical role in breaking down ECM barriers, releasing pro-migratory signals, and allowing for tumor cell invasion [8,9]. Use of pharmacological inhibitors such as marimastat is designed to block MMP activity [78]. These agents prevent ECM degradation thereby limiting tumor spread. Broad-spectrum MMP inhibitors have faced early challenges due to toxicities and insufficient efficacy; however, more recent studies have sought to refine this class of agents to improve selectivity and therapeutic windows by using nanoparticles [79,80].

Due to the role that MMPs play in driving breast cancer progression, leading to increases in ECM rigidity, several agents have been evaluated that target this class of enzymes. For instance, MMP-9 inhibitor (AB0046) targets HER2 amplified breast cancer in an immunocompetent mouse when used in combination with immunotherapy [81]. On the other hand, this agent blocked metastasis without appreciably impacting primary tumor growth in luminal B breast cancer [82]. MMP-14 inhibitor (DX-2400) demonstrated potent inhibition of primary tumor growth and lung and liver metastasis in an MDA-MB-231 orthotopic model of breast cancer [83]. DX-2400 also synergized with radiotherapy in preclinical breast cancer models [84]. Another MMP-14 inhibitor (Fab 3369) demonstrated promising anticancer activity in vitro and in vivo, disrupting the immune TME and reducing neoangiogenesis and hypoxia [85].

#### 4.5.3. Lysyl Oxidase (LOX) Inhibitors

Another important target is the enzymatic cross-linking of collagen mediated by lysyl oxidase (LOX) and its homologs, such as LOXL2 [6]. These enzymes can degrade dense ECM regions, thereby improving drug delivery and overcoming barriers to immune cell infiltration. LOX inhibitors such as β-aminopropionitrile showed promising activity in preclinical models of breast cancer [86,87,88]. In particular, targeting LOX has led to a reversal of chemoresistance in TNBC [89]. Because β-aminopropionitrile lacks sites for chemical modification, more work is needed to develop novel LOX inhibitors to treat breast cancer [90]. Antibody AB0023 represents another LOX inhibitor used to treat preclinical models of breast cancer and melanoma [91]. 4-methylumbelliferone (4-MU), a nontoxic dietary supplement, has been shown to reduce migration, adhesion, and invasion of breast cancer cells [92].

### 4.6. Agents That Target Mechanotransduction Pathways: Clinical Studies

A number of agents have undergone evaluation in clinical trials. Integrin antagonists have demonstrated promising activity, particularly when combined with other agents. For instance, integrin inhibitor cilengitide, when combined with paclitaxel, demonstrated promising results in a phase 1 trial, which included breast cancer patients (Table 1) [93]. This agent was well tolerated overall, though a few patients needed to discontinue therapy due to grade 4 dose-limiting toxicity of neutropenia. Another phase 1 clinical trial evaluated the antibody–drug conjugate sigvotatug vedotin (SGN-B6A) designed to target β6-integrin in solid tumors, including advanced HER2-negative breast cancer alone or in combination with pembrolizumab, with or without chemotherapy agents, cisplatin and carboplatin [94]. Recruitment for these clinical trials is ongoing.

Another clinical trial also confirmed that α5β3-integrin antagonist MEDI-522 was well tolerated overall in solid tumors, including those from patients with breast cancer though patients with renal cancer derived the most benefit [95]. MINT1526A (RG-7594) targets α6β1-integrin frequently overexpressed in solid tumors, including breast cancer and was evaluated in a phase I first-in-human open-label clinical trial. Though this agent was well tolerated, no further studies were pursued due to insufficient efficacy. Yet another phase I study entailed the use of GLPG-0187, a pan-integrin inhibitor, for advanced solid tumors, including breast cancer. This was a multicenter, open-label, dose-escalation, phase I study that found the drug was well tolerated but failed to demonstrate sufficient efficacy as a monotherapy, which reduced enthusiasm for its further development.

While targeting integrins holds more promise than directly targeting TGFβ signaling, a phase II trial initiated in 2002 used fresolimumab exclusively for patients with breast cancer and this agent directly targeted TGFβ signaling. This randomized controlled trial revealed that overall survival was improved with the higher dose of fresolimumab compared with the lower dose. Interestingly, the data reveal that patients exhibited dysfunctional T-cells compared with healthy controls, a phenomenon that was not overcome with fresolimumab, even when combined with radiotherapy. Despite this, these data suggest that TGFβ blockade can be optimal when used in combination with PD-1 blockade [96] (Table 1). The patients recruited in this trial demonstrated a history of progression on one or more therapies and had metastatic disease burden. The patient population was small. Interestingly, the FDA-approved angiotensin antagonist losartan is being evaluated in a phase II trial for breast cancer patients with radiation-induced fibrosis or who are at risk for developing this condition. This trial encourages the participation of female patients of all ancestries or ethnicities. Losartan has been shown to target TGFβ.

Lysyl oxidase (LOX) is a potential therapeutic target for a variety of malignancies, including colorectal and pancreatic cancer [97,98]. The copper chelator tetrathiomolybdate was evaluated in a phase II trial with a patient population of diverse ancestries (except African ancestry) at high risk of recurrence. Tetrathiomolybdate indirectly targets LOX. This agent was well tolerated overall and showed promising efficacy in patients with TNBC [99]. However, the trial was terminated early, and no plans are underway to re-initiate it.

Marimastat is an agent evaluated in phase III clinical trials for patients with metastatic breast cancer due to its capacity to target MMP-7 and MMP-9. This double-blind trial compared the effectiveness of marimastat with no further therapy for patients with metastatic breast cancer who responded or stabilized after initiation of chemotherapy. This was a large study involving over 300 patients over a 2-year period. However, MMP-7 and MMP-9 plasma levels were not predictive of patient response to this agent, and it did not significantly prolong progression-free survival [78,100].

Focal adhesion kinase (FAK) has proven to be a challenging target despite its relevance in ECM-mediated mechanotransduction in breast cancer. A phase II trial for FAK inhibitor IN10018 was initiated in March 2022 for patients with solid tumors, including TNBC. The primary target is FAK. This study is still recruiting, and results on the efficacy of IN10018 are therefore pending.

Pazopanib is an agent undergoing clinical trials that targets multiple mechanotransduction pathways, including the YAP/TAZ signaling pathway. Pazopanib was FDA-approved to treat advanced renal carcinoma in 2012. Patients with tumors that were hormone receptor-positive, HER2 receptor-negative, and FGFR1 amplified particularly responded to this agent [101]. Phase 1 clinical trials were initiated in patients with solid tumors, including breast cancer, in combination with MET/ALK/ROS1 inhibitor crizotinib [102]. This agent was well tolerated overall, with most adverse events being either grade 1 or 2 and reversible. However, this phase I trial was terminated due to the complexities of combination therapy.

Several mechanotransduction pathways become activated in response to changes in ECM stiffness, including the Src pathway. For instance, dasatinib and saracatinib have been previously evaluated in phase II clinical trials (Table 1). While saracatinib as a monotherapy demonstrated minimal efficacy [103], dasatinib demonstrated promising actions in breast cancer, particularly of the TNBC subtype [104,105]. To date, dasatinib is the only FDA-approved agent to treat chronic myeloid leukemia and acute lymphoblastic leukemia that is under evaluation for breast cancer and other solid malignancies [106].

PMD-026 is a first-in-class RSK inhibitor currently in phase II clinical trials to treat metastatic breast cancer, though it is historically used to treat myeloid malignancies [107,108]. Although it is considered a pan-RSK inhibitor, this agent shows slightly more selectivity for RSK2 over the other 3 family members [108]. RSK1 is mechanosensitive. This clinical trial is still recruiting patients.

### 4.7. Potential to Repurpose FDA-Approved Agents That Target ECM or Mechanotransduction Pathways

One efficient strategy to develop agents to treat breast cancer is to repurpose agents that target the ECM or mechanotransduction pathways that are FDA-approved for other medical conditions. Some FDA-approved agents for alternate conditions have demonstrated appreciable activity in breast cancer models in preclinical studies. For instance, antibodies against α4β1-integrin have been shown to counteract the transition from indolent micrometastasis to overt breast cancer metastasis [109]. Mechanotransduction mediated by α4-integrin has been primarily described with non-malignant conditions, such as inflammatory bowel disease [110,111,112]. Both vedolizumab and natalizumab are FDA-approved α4-integrin antagonists used to treat Crohn’s disease [113]. Pimozide, an FDA- approved drug for the treatment of Tourette’s syndrome, effectively inhibited breast cancer cell proliferation via targeting β1-integrin [114].

The YAP-TAZ pathway is the target for FDA-approved agents for other conditions that demonstrate benefit in the treatment of breast cancer. Verteporfin, an FDA-approved agent for use in the treatment of dry eye, promotes YAP-induced glycolysis in breast cancer cells [115]. Losartan, an FDA-approved agent that blocks angiotensin II receptors to promote blood pressure control, shows promise in breast cancer treatment [116] and we previously mentioned its current evaluation as a TGFβ blocker for breast cancer (Table 1). Losartan has been shown to block the oncogenic activity of YAP, thereby diminishing the growth of cholangiocarcinoma, a malignancy highly resistant to chemotherapy [117]. Dasatinib, though FDA-approved for the treatment of chronic myelogenous leukemia, demonstrates efficacy in breast cancer preclinical models, by inhibiting the LYN and YAP/TAZ pathways, particularly when used in combination [118,119]. Interestingly, atorvastatin, which is FDA-approved for the treatment of hypercholesterolemia via HMG-CoA reductase inhibition, has also been shown to inhibit YAP/TAZ signaling to restore chemosensitivity in chemoresistant breast cancer organoid models [120].

Other mechanotransduction pathways implicated in breast cancer progression may serve as potential agents to treat breast cancer. For instance, the AXL receptor tyrosine kinase inhibitor bemcentinib, an FDA-approved agent for the treatment of malignancies such as non-small cell lung carcinoma, was recently found to enhance sensitivity of anti-estrogens in breast cancer cells [121]. Pirfenidone, which is FDA-approved for idiopathic pulmonary fibrosis, primarily targets CAFs to curtail proliferation, metastases, drug resistance, and tumor stiffness in preclinical models of breast cancer [122]. Finally, hyaluronic acid-degrading hyaluronidase is an FDA-approved nano drug delivery system that can be used in conjunction with breast cancer therapy to help thwart cancer progression [123]. Hyaluronic acid degrades hyaluronidase, a major component of the ECM. A recent report demonstrated that hyaluronidase degradation synergized with photodynamic therapy to suppress breast cancer [124].

## 5. ECM Biomarkers and Clinical Implications

The ECM provides a rich source of biomarkers with significant diagnostic, prognostic, and therapeutic relevance in cancer [125]. Upcoming advances in detecting ECM-derived molecules and biochemical properties are changing their clinical utility, enabling noninvasive monitoring and directed personalized treatment strategies. In liquid biopsies, ECM-derived biomarkers, including soluble ECM components such as collagen fragments and proteoglycans are detectable in peripheral blood via immunoassays [125]. This offers a minimally invasive liquid biopsy method for assessing treatment responsiveness [125]. A great example of this would be PRO-C3, a fragment released during collagen III remodeling [6,125]. This fragment is elevated in metastatic breast cancer and colorectal cancer, correlating with poor prognosis. These biomarkers reflect the dynamic ECM remodeling and provide real-time insights into tumor progression and treatment response.

When considering therapeutic outcomes, it is crucial to look at biomechanical properties as biomarkers [125]. Alterations in ECM stiffness and architecture are critical determinants of therapeutic outcomes [126]. Beyond the soluble fragments, biochemical properties of the ECM critically influence breast cancer progression and therapeutic resistance. Increased stiffness resulting from excessive collagen cross-linking is a direct consequence of changes in ECM properties and can pose a challenge for new therapies [6]. Tumors with stiffer matrices primarily due to upregulation of LOX and related enzymes, readily promote cancer cell survival, invasion and metastasis [6]. It is worth noting that increased ECM stiffness can also modulate the TME, influencing the expression of immune checkpoint molecules such as PD-L1 and affecting the response to new immunotherapies [127]. Noninvasive imaging techniques, including ultrasound, elastography, and magnetic resonance elastography, are being explored in the clinical setting to assess ECM stiffness in breast tumors, offering additional tools for risk stratification and treatment planning [128,129].

There are many different clinical implications of ECM biomarkers in breast cancer [125]. On one hand, the ECM acts as a physical barrier that limits the penetration of chemotherapeutic agents and the infiltration of immune cells, contributing to treatment resistance. Furthermore, a remodeled ECM readily activates integrin-mediated signaling pathways [130,131]. A great example of this is β1-integrin/FAK signaling, which further enhances tumor cell survival and resistance to standard therapies [4,132]. These findings have prompted the development of therapeutic strategies that target the ECM directly. Inhibitors of ECM remodeling enzymes, including MMP inhibitors and LOX/LOXL2 inhibitors, are being revisited in combination with chemotherapy and immunotherapy to improve drug delivery and immune cell access [133,134,135]. Additionally, strategies to normalize the ECM using collagenase or integrin antagonists, such as cilengitide, are being explored for their potential to disrupt the pro-tumorigenic signaling and mechanical support provided by the ECM in breast cancer [4,6].

Unfortunately, despite these advances, several challenges remain. Current treatments for detecting ECM fragments in blood require further testing, and the heterogeneity of ECM composition across different breast cancer subtypes complicates the development of universal biomarkers [125]. Integrating ECM-derived biomarkers with genomic and proteomic profiling holds a lot of promise for more precise patient stratification and personalized therapy selection [125]. Patient stratification can be particularly critical when involving patients from diverse ancestry pools. The continued exploration of ECM biomarkers and their clinical applications in breast cancer is positioned to enhance early detection, improve prognostication, and help overcome barriers to effective treatment [125]. Though many agents with the capacity to target the ECM often showed insufficient efficacy when used as monotherapy, more promise was found with combination therapy. The repurposing of FDA-approved agents for other medical conditions deserves further exploration. This will pave the way for more individualized and efficacious breast cancer care.

## 6. Conclusions and Future Directions

**Figure 4:**
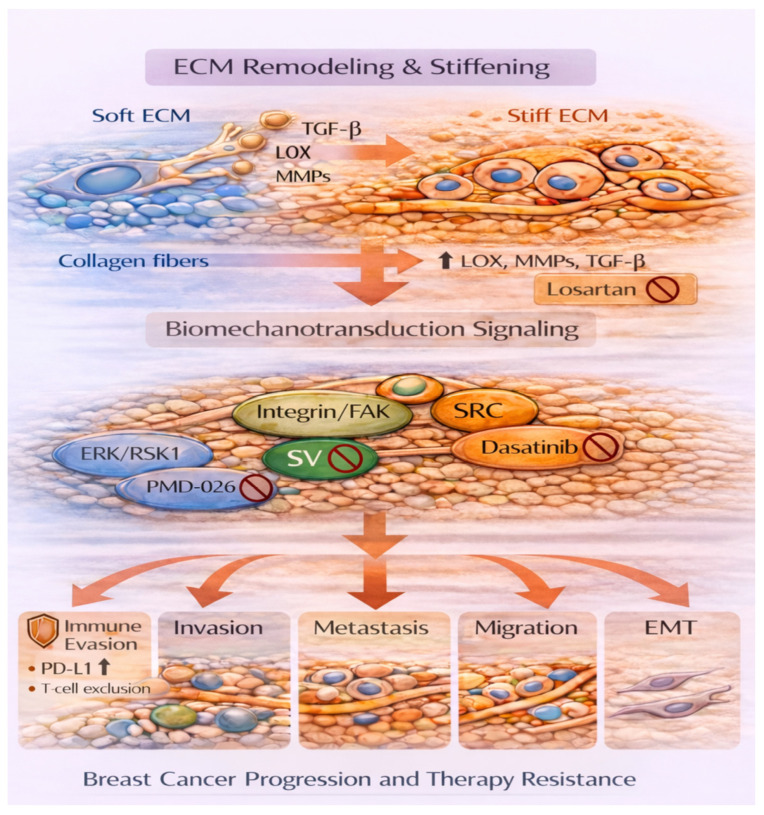
ECM stiffness-mediated activation of mechanotransduction pathways drives breast cancer progression and therapy resistance. Once the ECM converts to a stiff rather than a soft phenotype, this triggers activation of the mechanotransduction pathway, leading to immune evasion, invasion, migration, metastasis, and EMT. These processes then lead to breast cancer progression and therapy resistance. Losartan, which can block TGFβ; SV, which blocks β6-integrin; PMD-026, a RSK inhibitor; and dasatinib an SRC inhibitor, have the potential to thwart breast cancer progression and therapy resistance, to ultimately improve clinical outcomes among breast cancer patients. Abbreviations: programmed death ligand 1, PD-L1; sigvotatug vedotin, SV. Key: blue color, depicts soft ECM; orange color depicts stiff ECM, arrows, depict progression, small arrows, depict increase; 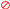, denotes agent-mediated inhibition of associated protein.

The ECM plays a central, dynamic role in cancer progression, actively shaping every stage (Figure 3 and Figure 4). The biochemical and biomechanical properties of the ECM influence tumor behavior, immune response, and treatment efficacy. Cancer cells exhibit abnormal ECM remodeling, which promotes metastasis, impedes drug delivery, and fosters therapeutic resistance, leading to poor outcomes. Targeting the ECM is an emerging therapeutic strategy that aims to disrupt tumor–ECM interactions to inhibit metastasis, enhance drug penetration, and overcome resistance. Current approaches include enzymatic degradation of ECM components, inhibition of cross-linking enzymes such as LOX, and blockade of integrin, TGFβ, RSK, and SRC signaling (Figure 4). However, given the ECM’s complexity and heterogeneity across breast cancer subtypes, therapies must be carefully designed and tailored to enhance their effectiveness.

Expanding patient databases to better represent diverse ancestries is essential for comparative analyses, biomarker discovery, and personalized risk assessment [136]. Because genetic background influences ECM structure and function, precision oncology in breast cancer should move beyond one-size-fits-all strategies and instead tailor therapies that consider ECM remodeling patterns. This shift in therapeutic approaches requires ECM-based preclinical models derived from patients of diverse ancestries and greater inclusion of underrepresented populations in clinical trials [136]. Although many trials reviewed here did not report patient ancestry, ongoing efforts to recruit more diverse cohorts are encouraging and are expected to help reduce breast cancer disparities.

Advances in biomarker discovery and imaging technologies should help drive the development of ECM-targeted therapies [125]. ECM-derived fragments and remodeling enzymes detectable within patient samples hold promise as minimally invasive diagnostic and prognostic markers, as well as tools for monitoring therapeutic response and improving patient stratification.

In summary, the ECM is an active driver of breast cancer progression, and targeting mechanotransduction pathways represents a promising frontier in precision oncology. While no FDA-approved ECM- or mechanotransduction-targeted therapies exist to date, ongoing clinical trials and opportunities for drug repurposing are encouraging. Continued interdisciplinary and translational research will be critical to developing selective, context-specific ECM modulators and integrating these ECM-targeted strategies with standard-of-care therapies to treat breast cancer. A deeper understanding of therapy-induced ECM remodeling and its impact on tumor behavior will be essential for optimizing combination treatments, limiting resistance, and ultimately improving outcome equity for patients with breast cancer.

## Figures and Tables

**Table 1:** Clinical trials involving agents that target extracellular matrix and mechanotransduction signaling pathways.

Agent(s)	Patient Population	Primary Endpoint	Secondary Endpoint	NCT No.	Target	Patient Ethnicity/Ancestry	Trial Location (Country)	Status *
Cilengitide with Paclitaxel	Advanced solid tumors (including breast cancer)	AE, CR, DLT, OS, PFS, PR	TEAE, PK	NCT01276496	α5β3 − integrin α5β5 − integrin	European 83.3% African 17.7%	United States	Phase I: Completed
SGN-B6A (sigvotatug vedotin) alone or with Pembrolizumab, or with chemotherapy	Advanced solid tumors (including HER2— breast cancer)	AE, DLT	ADA, DOR, ORR, OS, PFS, PK	NCT04389632	β6-Integrin	Patients of any ancestry/ethnicity eligible and encouraged to participate	United States, France, South Korea, Spain, Switzerland, Taiwan, United Kingdom	Phase I: Ongoing/ Recruiting
MEDI-522	Advanced malignancies including breast cancer	MTD, safety, tolerability	SD, ORR	NCT00049712	α5β3 − integrin	Unspecified	United States	Phase I: Completed
MINT1526A as single agent and in combination with bevacizumab	Advanced solid tumors including breast cancer	DLT, SAE, safety	PK, CL	NCT01139723	α6β1 − integrin	Unspecified	United States	Phase I: Completed
GLPG-0187	Advanced solid tumors (including breast cancer)	DLT, Safety, tolerability	PK, PE	NCT01313598	Integrin receptors	Unspecified	Netherlands	Phase I: Completed
Fresolimumab + radiotherapy	Metastatic breast cancer	Abscopal response rate	-	NCT01401062	TGF-β	Unspecified	United States	Phase II: Completed
Losartan	Invasive breast cancer	Reduction in radiation-induced fibrosis	Change in breast volume, improved cosmesis	NCT05637216	TGF-β	Women of any ancestry/ethnicity eligible and encouraged to participate	United States	Phase II: Ongoing/ Recruiting
Tetrathiomolyb-date, a copper chelator	Breast cancer with high risk for recurrence	TTP	-	NCT00195091	LOX	European: 80% Hispanic ethnicity: 12.5% Asian: 5% Other: 1% African: 0%	United States	Phase II: Terminated
Marimastat	Metastatic breast cancer	PFS	PFS	NCT00003010	Matrix metalloproteinases 7 & 9	Unspecified	United States	Phase III: Completed
IN10018 + doxorubicin (liposomal) +/− Toripalimab	Solid tumors including metastatic TNBC	ORR, CR, PR	CR, PR, SD	NCT05830539	FAK	Unspecified	China	Phase Ib/II ongoing/recruiting
Pazopanib with Crizotinib or Pemetrexed; or various combinations	Advanced solid tumors (included breast cancer)	MTD	-	NCT01548144	MAPK/ERK, YAP/TAZ	Unspecified	United States	Phase I: Terminated
Saracatinib	HR—metastatic breast cancer	Disease control rate	ORR, PFS	NCT00559507	Src kinase	Unspecified	United States	Phase II: Completed
Dasatinib in combination with paclitaxel	Metastatic breast cancer	ORR	CR, PR	NCT01042535	Src kinase	Unspecified	United States	Phase II: Completed
Dasatinib in combination with paclitaxel	Metastatic HER2+ breast cancer	MTD, ORR	CP, TTP	NCT01306942	Src kinase	Unspecified	Spain	Phase I/II: Completed
PMD-026	Metastatic breast cancer	AE, DLT, SAE, Safety, tolerance	DCR, DOR, ORR, PFS, PK	NCT04115306	RSK (p90 ribosomal S6 kinase) family of proteins	Unspecified	United States	Phase II: Ongoing/ Recruiting

Abbreviations: ADA, antidrug antibody; AE, adverse event; CR, complete response; CB, clinical benefit, CL, clearance; Cmax, maximum plasma concentration; DCR, disease control rate; DLT, dose-limiting toxicity; DOR, duration of response; MTD, maximum tolerated dose; OPD, optimal pharmacokinetic dose; ORR, overall/ objective response rate; OS, overall survival; PE, preliminary efficacy; PK, pharmacokinetic parameters; PR, partial response; SAE, serious adverse events; SD, stable disease; TEAE, treatment-emergent adverse events, TTP, Time to progression. * As of 1 January 2026.

## Data Availability

No new data were created or analyzed in this study. Data sharing is not applicable to this article.
